# Behavioural determinants influencing continuing professional development in healthcare practice: a qualitative study

**DOI:** 10.1007/s11096-025-02079-8

**Published:** 2026-01-16

**Authors:** Heba Al-Omary, Abderrezzaq Soltani, Derek Stewart, Zachariah Nazar

**Affiliations:** 1 College of Pharmacy, QU Health, Qatar University, Doha, Qatar; 2 Office of Vice President for Health and Medical Sciences, QU Health Sector, Qatar University, Doha, Qatar

**Keywords:** Behavior change, Continuing education, Health personnel, Implementation, Professional practice

## Abstract

**Introduction:**

The integration of learning from continuing professional development (CPD) activities into practice is shaped by behavioral, organizational, and broader system-level factors. However, there is scarce research utilizing theory to provide a comprehensive understanding of prevalent behavioral determinants.

**Aim:**

To investigate key behavioral determinants that influence CPD participants’ implementation of learning into their practice following participation in CPD activities.

**Method:**

Eleven semi-structured interviews were conducted with healthcare professionals 4–6 weeks after they participated in a live, interactive CPD workshop. Interview questions were guided by the COM-B model to elucidate behavioral determinants; emerging themes were subsequently mapped to the COM-B domains. Recommended interventions were derived to optimize CPD outcomes using the Behavior Change Wheel (BCW).

**Results:**

All participants (n = 11) reported applying their CPD learning in practice, either partially or fully). Analysis revealed that while *Capability, Opportunity*, and *Motivation* were all perceived to influence implementation, *Motivation* was an important driver, with professional responsibility and satisfaction from positive patient outcomes were also perceived to influence behavior. *Opportunity* was particularly challenging in community pharmacy settings due to time constraints, workload, and organizational factors. These findings informed targeted recommendations to optimize CPD implementation.

**Conclusion:**

This study highlights the complex interplay of behavioral determinants that are perceived to influence the translation of CPD learning into routine clinical practice. Effective CPD programs should incorporate strategies to address setting-specific barriers—such as time constraints, emotional pressures, and organizational support to foster motivation and facilitate sustained practice change. Tailoring CPD design to these behavioral determinants can improve the integration of learning into practice and ultimately enhance patient care.

## Impact statements

- CPD programs should move beyond knowledge delivery by creating opportunities for reflection, patient-centered counseling practice, and peer discussion to support real-world application.
- Organizational and managerial backing, including protected time, supportive workplace culture, and recognition of CPD efforts, is critical for successful implementation.
- Patient engagement strategies (e.g., building trust, tailoring education to literacy and language needs) should be emphasized within CPD design and follow-up.
- Intrinsic motivators such as professional responsibility and personal satisfaction can be leveraged by aligning CPD content with professionals’ values and sense of duty.
- Local economic and resource contexts should be acknowledged, as commercial pressures may either support or undermine CPD implementation.

## Introduction

Continuing Professional Development (CPD) is an ongoing process through which healthcare professionals maintain and enhance their knowledge, skills, and competencies to meet evolving clinical standards and ensure safe and effective patient care [1, 2]. Beyond developing technical expertise, CPD fosters personal and professional growth, enabling providers to deliver high-quality services that meet the complex needs of patients and communities [2–4]. A growing body of evidence has consistently shown that CPD contributes to healthcare quality improvement and patient outcomes by facilitating the adoption of evidence-based practices and supporting knowledge translation into clinical care [5].

Although CPD has demonstrated benefits, its impact on routine clinical practice is often inconsistent. Healthcare professionals may struggle to apply newly acquired knowledge and skills due to barriers such as time constraints, limited resources, and lack of organizational support [5] Implementation science emphasizes that knowledge acquisition alone does not ensure practice change or improved patient outcomes [6]. Successful implementation requires integration of evidence-based interventions into real-world settings, along with reflection on how new learning shapes care delivery [7, 8]. Reviews further highlight the influence of individual attitudes, competing demands, organizational culture, and economic considerations, though methodological variability limits generalizability and underscores the need to understand the mechanisms enabling successful implementation [9, 10].

The integration of CPD learning into practice is shaped by behavioral, organizational, and broader system-level factors. At the behavioral level, active engagement is needed, as passive participation rarely leads to change [11, 12]. At the organizational level, factors such as workplace culture and managerial support can facilitate or hinder implementation [13, 14]. At the system level, issues such as national policies, healthcare financing, and regulatory structures can constrain the sustainability of CPD impact. A recent scoping review highlighted the limited understanding of how these layers interact and emphasized the need for CPD initiatives to support both individual behavior change and wider system improvements [15]

Despite the numerous studies to report on the barriers that hinder the implementation of skills and knowledge from CPD activities, much of this research has been largely descriptive and not grounded in theory [16, 17]

The COM-B model is particularly useful in this context, as it conceptualizes behavior as the interaction of Capability (knowledge and skills), Opportunity (environmental and social context), and Motivation (goals, emotions, and beliefs) [18]. Applying COM-B to CPD implementation allows for comprehensive identification of barriers and facilitators, while linking them to evidence-based behavior change techniques [19, 20]. This model has been successfully applied to diverse health behaviors such as medication adherence, physical activity, and healthy eating [21–25], but its use in CPD implementation research remains limited. Leveraging behavioral theory in this field therefore represents an important opportunity to generate deeper understanding and develop interventions that are both meaningful and sustainable [18, 26]

### Aim

This study utilizes an implementation science approach to explore the barriers and facilitators to applying CPD-acquired knowledge in clinical settings. Focusing on a CPD workshop on asthma care in Qatar, the study applies the COM-B model to systematically identify determinants of practice change.

Research question: What are the key behavioral determinants that influence CPD participants’ implementation of learning into their practice?

## Method

### Study design

This study employed a qualitative descriptive design.

#### Participants selection and sampling

Participants were licensed pharmacists and nurses practicing in Qatar with regular clinical contact with asthma patients, who attended one of two CPD asthma workshops held between November 2023 and January 2024. Following the workshop, attendees received an information sheet outlining the study objectives and procedures, and a form seeking consent to be contacted for interviews. Consenting participants were invited for one-on-one interviews 4–6 weeks post-workshop, allowing sufficient time to reflect on and apply learning in practice.

Participation was therefore based on an open invitation, and the final group reflected a self-selected sample rather than purposive recruitment. Recruitment continued with follow-up email invitations sent 2–4 weeks after the initial invitation, until informational redundancy was reached. This was defined as the point at which additional interviews yielded no substantively new insights, consistent with established qualitative research guidance [27].

#### Development of data collection tools

The semi-structured interview guide was developed following a five-phase process [28] involving the identification of prerequisites, literature review, preliminary formulation, pilot testing, and finalization. The guide was explicitly aligned with the COM-B model to capture behavioral determinants influencing implementation of learning.

To enhance the credibility and trustworthiness of the interview guide, it was reviewed by three local experts in behavioral science and qualitative research. Two pilot interviews were conducted with a practicing pharmacist and one with a nurse to refine question clarity, flow, and comprehensiveness, ensuring participants could engage meaningfully and provide rich data.

### Data collection

Data were collected through one-on-one, semi-structured interviews, conducted either in person or via secure Microsoft Teams, depending on participant preference. Either the principal investigator (ZN) or the primary researcher (HAO) led the interviews with the other always present to support the process. Face-to-face interviews were conducted in the private office of the principal investigator at Qatar University. Interviews were audio-recorded with informed consent and lasted approximately 30–45 min, as indicated in the participant information sheet. Brief field notes and reflective memos were documented during and after each interview to capture contextual details, initial impressions, and emerging ideas, supporting the trustworthiness of the data.

The primary researcher (HAO) had formal training in qualitative research and semi-structured interviewing, including coursework and workshops addressing ethical considerations, interview skills, and data management. Supervision and methodological oversight were provided by an experienced qualitative researcher (ZN) throughout the data collection process. Researchers had no prior relationships with participants, minimizing potential bias and enhancing the independence of data collection.

### Data analysis

A deductive–inductive framework analysis approach was applied, using the COM-B model as an overarching analytical framework to systematically organize participant experiences in relation to established theoretical constructs [29]. Audio recordings were transcribed verbatim using Microsoft Teams transcription tools and verified for accuracy by the primary researcher (HAO).

In‑person interviews were audio‑recorded on a digital recorder and transcribed verbatim by the primary researcher (HAO), with a second reviewer (ZN) verifying accuracy against the audio and removing identifiers prior to analysis.

Coding was conducted in Microsoft Excel using color-coding to differentiate categories and themes.

1. Preliminary Coding: Two researchers (ZN, HAO) conducted primary coding that combined both deductive and inductive elements. Initially, transcripts were read openly to capture all relevant concepts emerging from participants’ narratives (i.e., open coding). Each meaning unit was then examined to determine whether it reflected a facilitator or barrier and was subsequently linked deductively to one of the COM-B components—Capability, Opportunity, or Motivation. A provisional codebook was developed from both the interview guide and emergent data and refined iteratively through joint review.
2. Sub-theme Identification: Related codes were grouped and refined into sub-themes that aligned with COM-B subcomponents (e.g., physical vs. psychological capability; social vs. physical opportunity; reflective vs. automatic motivation). While the overarching categorization followed COM-B deductively, the specific codes and sub-themes were inductively derived from participants’ own descriptions.
3. Integration and Verification: Discrepancies in coding and categorization were discussed and resolved through consensus among the research team (HAO, ZN, DS, AS), ensuring both trustworthiness and conceptual alignment with the COM-B framework. The finalized matrix of COM-B components and sub-themes represented the validated framework of behavioral determinants influencing CPD implementation.
4. Application of the Behavior Change Wheel (BCW): Building on the finalized COM-B framework, the Behavior Change Wheel was then applied to translate these determinants into actionable intervention strategies. Behavioral factors under each COM-B domain were systematically mapped to relevant BCW intervention functions, such as education, training, persuasion, incentivization, environmental restructuring, and modeling, following the process described by Michie et al. (2011) [18]. This step enabled the transformation of qualitative insights into theory-informed recommendations for optimizing CPD design and implementation.

### Trustworthiness and rigor

Several strategies were employed to enhance rigor and ensure credibility as advocated by Shenton [30].

- Maintaining an audit trail documenting all research steps.
- Conducting inter-coder reliability checks to ensure consistency in coding. This was achieved through an iterative process in which two researchers (ZN and HAO) independently coded a subset of transcripts (approximately 20%) and then compared coding outputs to assess consistency in code application and theme interpretation. Discrepancies were discussed in detail to achieve consensus, and the refined code definitions were integrated into an updated codebook, which was subsequently applied to the remaining transcripts.
- Engaging in regular peer review with supervisors.
- Keeping comprehensive records of transcripts, field notes, reflective memos, and coding decisions.

### Ethics approval

Ethical approval was obtained in November 2023 from the Institutional Review Board (IRB) at Qatar University [QU-IRB 1985-EA/23].

## Results

### Demographic characteristics

Seventy-six healthcare professionals (HCPs) attended the asthma care workshop, all of whom were contacted afterward and invited to participate in the study. Initially eight HCPs agreed to participate; however, recruitment continued with two follow-up invitations until informational redundancy was achieved. A total of 11 healthcare professionals (HCPs) participated in the interviews, including 8 pharmacists and 3 nurses. The participants represented a range of healthcare settings: 5 from hospitals, 2 from community pharmacies, 3 from private primary healthcare facilities, and 1 from a governmental primary healthcare center. Eight participants were female, and three were male. The majority (7 participants) had between 5 and 10 years of professional experience. Participants held various professional roles, including clinical pharmacists, community pharmacists, and specialized nurses, ensuring a diverse representation of perspectives (Table 1).

**Table 1:** Participants’ demographic characteristics

Participant ID	Profession	Healthcare Setting	Gender	Years of experience
P1	Pharmacist	Community Pharmacy	Male	5–10
P2	Pharmacist	Governmental hospital	Female	3–5
P3	Pharmacist	Governmental hospital	Female	5–10
P4	Pharmacist	Community Pharmacy	Male	10–15
P5	Nurse	Private home care service	Male	5–10
P6	Pharmacist	Governmental hospital	Female	3–5
P7	Nurse	Private medical center	Female	5–10
P8	Pharmacist	Private hospital	Female	5–10
P9	Nurse	Governmental Primary health care center	Female	10–15
P10	Pharmacist	Governmental hospital	Female	5–10
P11	Pharmacist	Governmental hospital	Female	5–10

### Qualitative themes and codes mapped to COM-B components

All the interviewed participants indicated that they were able to implement their learning from the CPD activity into their practice either in part or fully. Analysis of the qualitative interviews identified seven overarching themes reflecting the facilitators and barriers experienced by healthcare professionals when implementing CPD learning into practice. These themes were systematically mapped to the COM-B model components (Capability, Opportunity, and Motivation) to provide a structured understanding of behavioral determinants. The mapping also informed potential intervention strategies using the Behavior Change Wheel (BCW), highlighting how individual, social, and organizational factors interact to influence the translation of learning into clinical practice.

Table 2 presents the identified themes, its associated codes, and the corresponding COM-B components.

**Table 2:** Qualitative Themes with Codes Mapped to COM-B Components and Their Influence (Facilitators or Barriers)

Theme	Code	COM-B Component (Sub-themes)	Facilitator (✔) /Barrier (X)
Knowledge and Skills for Implementing CPD Learning	Practical Skills	Physical Capability	✔
	Previous Experience and Expertise	Physical Capability	✔
	Need for Additional Skills	Physical Capability	X
	Acquired Knowledge from CPD	Psychological Capability	✔
	Knowledge Gaps Pre-CPD	Psychological Capability	X
Time Constraints and Competing Workload Demands	Prioritizing Key Information	Psychological Capability	✔
	Lack of Time for Counseling	Physical Opportunity	X
	Rush Hours	Physical Opportunity	X
	Time Management Challenges	Psychological Capability	X
Engaging and Educating Patients in Asthma Care	Patient Feedback	Automatic Motivation	✔
	Building Trust	Social Opportunity	✔
	Personalized Counseling	Psychological Capability	✔
	Patient Willingness	Social Opportunity	X
	Language Barriers	Physical Opportunity	X
	Patient Cognition	Psychological Capability	X
Organizational and Managerial Support Systems	Management Support	Social Opportunity	✔
	Colleague Encouragement	Social Opportunity	✔
	Lack of Incentives	Automatic Motivation	X
	Disinterest from Colleagues	Social Opportunity	X
Professional Responsibility and Accountability	Obligation to patient care	Reflective Motivation	✔
	Ethical commitment	Reflective Motivation	✔
	Management’s focus on profit	Reflective Motivation	X
Economic and Resource Considerations	Increased profit through compliance	Social Opportunity	✔
	Trust Building Leads to Business Growth	Social Opportunity	✔
	Profit over Patient Care	Reflective Motivation	X
Personal Satisfaction and Professional Motivation	Professional Satisfaction	Reflective Motivation	✔
	Positive Feedback from Patients	Automatic Motivation	✔
	Sense of Achievement	Reflective Motivation	✔
	Emotional Pressure	Automatic Motivation	X

#### Theme 1: Knowledge and Skills for Applying CPD Learning

Participants described how CPD workshops improved their knowledge and counseling skills, which supported effective asthma care (facilitator). For example, one nurse shared: *“So together, the patient and I assess… they are more self-aware now than before, so that was the good thing I gained with this workshop.”* (P7, Nurse). However, gaps remained, with some reporting limited confidence in managing complex cases (barrier): *“Now I can share my knowledge with them… but I need more experience when dealing with children with asthma.”* (P9, Nurse).

#### Theme 2: Time Pressures and Competing Workload Demands

Heavy patient loads and long shifts restricted opportunities for in-depth counseling (barrier). As one pharmacist explained: *“Sometimes because we have rush hours in the community pharmacy… during those times, it’s hard to find time for in-depth counseling.”* (P1, Pharmacist). At the same time, participants highlighted strategies to prioritize essential information and streamline consultations (facilitator): *“It organized my line of thoughts… What will I address first about asthma?”* (P2, Pharmacist).

#### Theme 3: Patient Engagement and Counseling Approaches

Building trust, tailoring information, and responding to patient feedback encouraged adherence (facilitator): *“So when I am counseling and using the chart, they think that I am caring… We have a trust, and they will improve and come back.”* (P1, Pharmacist). Yet, engagement was sometimes hindered by patient disinterest, language barriers, or limited health literacy (barrier): *“It depends on the patient… Some patients don’t like comprehensive information and aren’t interested in listening.”* (P2, Pharmacist).

#### Theme 4: Organizational and Managerial Support

Supportive workplace cultures and peer collaboration were seen as enablers: *“Applying new steps… we educate each other more. This supports the enhancement of care delivery overall.”* (P11, Pharmacist). In contrast, absence of managerial backing, lack of incentives, or unsupportive environments—particularly in private settings—restricted implementation (barrier): *“This is the private sector… There is no support.”* (P8, Pharmacist).

#### Theme 5: Professional Responsibility and Ethical Duty

A strong sense of professional obligation motivated participants to integrate asthma education into care (facilitator). As one nurse reflected: *“It makes me feel like being a nurse is not only a job, but there is a bigger mission with this job.”* (P7, Nurse). However, this was undermined when organizational priorities emphasized profit over patient education (barrier): *“In the private sector, there is no encouragement… It’s a business, it’s commercial.”* (P8, Pharmacist).

#### Theme 6: Economic and Resource Considerations

Financial drivers operated in both directions. On one hand, trust-building and effective education increased adherence, benefiting both patients and organizations (facilitator): *“I have my profit to increase my sales, but at the same time, I am building trust with my patients.”* (P1, Pharmacist). Conversely, a profit-first mindset reduced emphasis on patient education (barrier).

#### Theme 7: Personal Satisfaction and Professional Motivation

Professional fulfillment, confidence, and patient loyalty reinforced the value of applying CPD learning (facilitator). One pharmacist explained that seeing a patient’s symptoms improve after counselling “reminded me why I chose this profession—it’s satisfying when you know your advice really helped” (P4, Pharmacist). Another participant shared that patient appreciation and follow-up visits “made me feel proud and eager to keep improving” (P3, Pharmacist). However, participants also acknowledged that this motivation could be challenged by demanding workloads and emotionally taxing cases, especially when treatment progress was slow or uncertain (barrier). As one nurse reflected, “sometimes when the patient doesn’t respond as expected, you start doubting yourself for a moment—but you keep learning and trying again” (P5, Nurse).

Collectively, these seven themes illustrate the complex interplay of capability, opportunity, and motivation in shaping healthcare professionals’ ability to implement their CPD learning into practice. While facilitators such as enhanced knowledge and skills, patient trust, professional responsibility, and personal satisfaction were strongly emphasized, these were frequently offset by barriers including time constraints, lack of systemic support, and competing economic priorities.

### Mapping COM-B components to proposed interventions via the behavior change wheel

Table 3 shows how the COM-B components identified in the qualitative data were mapped to the Behavior Change Wheel (BHW) which informed targeted intervention strategies.

**Table 3:** Mapping of Themes and Codes to COM-B Components, and Proposed Behavior Change Wheel (BCW) Intervention Functions

Theme	COM-B Component	Intervention Function (BCW)
Knowledge and Skills	Capability (physical/psychological)	Education, Training e.g.. Routinely incorporate interactive, skills-based CPD strategies (e.g., simulation, case-based learning, supervised practice)
Time Constraints	Opportunity (physical), Capability (psychological)	Environmental restructuring, Training
Patient Engagement	Opportunity (social), Capability (psychological), Motivation (automatic)	Social support, Persuasion, Modeling
Support Systems	Opportunity (social), Motivation (automatic)	Environmental restructuring, Social support, Incentivization
Professional Responsibility	Motivation (reflective)	Persuasion, Modeling
Economic considerations	Opportunity (social), Motivation (reflective)	Incentivization, Persuasion
Personal Satisfaction	Motivation (reflective & automatic)	Reinforcement, Modeling

Capability-related factors (e.g., knowledge and skills gaps) were linked to interventions focused on education and training. Opportunity-related factors (e.g., time constraints, management support, peer encouragement) aligned with recommendations for environmental restructuring and social support. Motivation-related factors (e.g., ethical commitment, profit motives, personal satisfaction) mapped to recommended interventions including persuasion, incentivization, reinforcement, and modeling.

## Discussion

### Summary of key findings

This study explored perceived behavioral determinants influencing HCPs ability to implement learning from an asthma CPD workshop into practice. Eleven participants (pharmacists and nurses) contributed perspectives across hospital, community, private, and primary care settings.

The analysis identified seven themes that reflected both facilitators and barriers to implementation. While CPD enhanced knowledge and skills, external pressures such as time constraints, lack of incentives, and unsupportive colleagues limited application. Conversely, professional responsibility, patient engagement, and personal satisfaction served as intrinsic motivators.

Linking these findings to COM-B revealed that capability was shaped by knowledge and skills as well as time management, opportunity was influenced by organizational and social contexts, and motivation was driven by intrinsic factors (ethical commitment, satisfaction) and extrinsic barriers (profit focus, limited incentives).

Overall, the findings illustrate that while CPD successfully builds capability, sustainable implementation requires supportive opportunities and reinforced motivation at both individual and organizational levels.

### Interpretation of findings and implications

This study demonstrates that implementing CPD learning is shaped by the interplay of capability, opportunity, and motivation, rather than knowledge alone. While participants reported that CPD enhanced their knowledge and confidence, the sustainability of practice change was influenced by contextual and motivational factors.

Capability was strengthened through knowledge acquisition and improved confidence in patient counseling. Similar findings have been reported in CPD research with midwives [31] and with dietetic counseling for cancer survivors [32]. However, gaps in practical skills limited translation into practice, echoing calls for CPD programs to incorporate hands-on training to ensure that HCPs can apply knowledge in real-world settings [33]. This finding supports recommendations that CPD should go beyond information delivery and include interactive, skills-based components such as simulation, case-based learning, and supervised practice to strengthen physical capability [34–37]

Opportunity, particularly time and workload, was a commonly cited barrier. This aligns with the *Environmental Context and Resources* domain, where limited time and staffing consistently undermine implementation [38, 39] Social opportunity also played a pivotal role: managerial and peer support enabled change, while disengaged colleagues and unsupportive leadership hindered it. These findings are consistent with prior research showing that organizational culture and leadership support are essential for embedding evidence-based practice [31, 32].To maximize CPD impact, organizational leaders must address systemic barriers such as workload distribution, provide formal recognition or protected time for learning, and foster peer networks that normalize new practices.

Automatic motivation was often undermined by emotional pressures, including stress and patient demands, reflecting the *Emotion* domain [32, 40] Yet reflective motivation, tied to professional responsibility and ethical duty, was also a facilitator, consistent with evidence that professional identity and beliefs about consequences are critical for sustained behavior change [18, 39] Patient feedback provided reinforcement that strengthened motivation, but patient resistance or language barriers acted as deterrents, highlighting the complexity of interpersonal dynamics [31]. These findings suggest that CPD should explicitly address motivational factors by incorporating strategies to build resilience, enhance communication skills, and reinforce professional identity. At the organizational level, systems of feedback, recognition, and incentivization can help sustain motivation and counteract emotional fatigue.

Economic considerations had a dual influence. In some settings, financial incentives reinforced implementation. However, when profit motives conflicted with patient care, they undermined intrinsic motivators such as professional responsibility [41]. To address this, policymakers and healthcare organizations should carefully balance the use of financial incentives with measures that support professional values, ensuring that commercial priorities do not erode patient-centered care.

Overall, these findings reinforce that CPD can effectively build capability, but lasting impact depends on addressing opportunity and motivation through multilevel interventions. CPD design should therefore adopt a systems perspective, integrating educational content with organizational and policy-level strategies that tackle barriers and reinforce facilitators.

### Strengths and limitations

A notable strength is the theoretical grounding of the study. The use of the COM-B model provided a systematic framework for data collection, analysis, and interpretation, ensuring that findings were not only descriptive but also mapped to established behavior change theory. This enhanced the credibility of the findings and strengthened their potential to inform targeted interventions via the BCW. The study also benefited from qualitative depth, capturing barriers and facilitators across diverse healthcare settings, which supports confirmability by grounding interpretations in rich participant accounts.

Limitations include the small sample size, which may not reflect the perspectives of all healthcare professionals and therefore constrains transferability beyond similar contexts. Self-selection bias is possible, as those more motivated or engaged with CPD may have been more likely to participate, potentially under-representing less engaged individuals. The gender imbalance in the sample, with fewer male participants, may also have limited the diversity of perspectives. Finally, as a local study based on a single CPD activity, the findings may not generalize to other professional groups, specialties, or national settings. These factors should be considered when interpreting the results. Nonetheless, by applying theory and drawing on diverse practice settings, the study offers insights with practical relevance and contributes to the trustworthiness of evidence on CPD implementation.

### Recommendations for future studies

Future research should extend beyond individual healthcare professionals to include the perspectives of administrators, managers, and policymakers. Understanding how organizational culture, leadership, and resource allocation influence CPD implementation could help identify structural changes needed to address barriers such as workload, time pressure, and lack of support.

Building on this study, there is also a need to design and test tailored interventions that target specific determinants of behavior using the COM-B model. Potential strategies include time-management tools, stress-reduction initiatives, workload adjustments, and peer-support mechanisms to strengthen both opportunity and motivation for applying CPD learning.

Finally, to enhance generalizability, future studies should adopt mixed-methods approaches. Large-scale surveys could establish the prevalence of identified barriers and facilitators across different settings, while qualitative interviews or focus groups would add depth and contextual understanding. Such approaches would validate these findings and support the development of scalable, evidence-informed strategies for CPD implementation in diverse healthcare environments.

## Conclusion

This study contributes new evidence on the behavioral determinants influencing the implementation of CPD learning, highlighting that its success depends not only on enhanced knowledge and skills but also on addressing motivational and organizational factors. Motivation—through professional responsibility, patient engagement, and personal satisfaction—was a strong facilitator, while opportunity-related barriers such as time constraints, workload, and limited management support hindered change. These findings emphasize that CPD initiatives must move beyond knowledge delivery to incorporate strategies that strengthen opportunity and reinforce motivation.

By applying the COM-B model, this research provides a theory-driven framework to guide the design of targeted interventions, with the BCW offering practical solutions for addressing barriers and enhancing facilitators. For healthcare organizations, aligning CPD with supportive structures and incentives can promote sustained behavior change, leading to improved patient care and the advancement of healthcare practice.

## Data Availability

All data supporting the findings of this study are provided in the results section of the article.
